# Untargeted Metabolomics Toward Systematic Characterization of Antioxidant Compounds in Betulaceae Family Plant Extracts

**DOI:** 10.3390/metabo9090186

**Published:** 2019-09-16

**Authors:** Sunmin Lee, Dong-Gu Oh, Digar Singh, Hye Jin Lee, Ga Ryun Kim, Sarah Lee, Jong Seok Lee, Choong Hwan Lee

**Affiliations:** 1 Department of Bioscience and Biotechnology, Konkuk University, Seoul 05029, Korea; duly123@naver.com (S.L.); dhehdrn@konkuk.ac.kr (D.-G.O.); singhdigar@gmail.com (D.S.); 2 Department of Biological Resources Utilization, National Institute of Biological Resources, Environmental Research Complex, Incheon 22755, Korea; zzinni87@naver.com (H.J.L.); ryun31@korea.kr (G.R.K.); lsr57@korea.kr (S.L.); jslee001@korea.kr (J.S.L.); 3 Research Institute for Bioactive-Metabolome Network, Konkuk University, Seoul 05029, Korea

**Keywords:** Betulaceae, *Alnus firma*, mass spectrometry, bioassays, ethyl gallate

## Abstract

Plant species have traditionally been revered for their unparalleled pharmacognostic applications. We outline a non-iterative multi-parallel metabolomic-cum-bioassay-guided methodology toward the functional characterization of ethanol extracts from the Betulaceae family plants (*n* = 10). We performed mass spectrometry (MS)-based multivariate analyses and bioassay-guided (ABTS antioxidant activity and cytoprotective effects against H_2_O_2_-induced cell damage) analyses of SPE fractions. A clearly distinct metabolomic pattern coupled with significantly higher bioactivities was observed for 40% methanol SPE eluate. Further, the 40% SPE eluate was subjected to preparative high-performance liquid chromatography (prep-HPLC) analysis, yielding 72 sub-fractions (1 min^−1^), with the highest antioxidant activities observed for the 15 min and 31 min sub-fractions. We simultaneously performed hyphenated-MS-based metabolite characterization of bioactive components for both the 40% methanol SPE fraction and its prep-HPLC sub-fraction (15 min and 31 min). Altogether, 19 candidate metabolites were mainly observed to contribute toward the observed bioactivities. In particular, ethyl gallate was mainly observed to affect the antioxidant activities of SPE and prep-HPLC fractions of *Alnus firma* extracts. We propose an integrated metabolomic-cum-bioassay-guided approach for the expeditious selection and characterization of discriminant metabolites with desired phenotypes or bioactivities.

## 1. Introduction

Plant-derived natural products, i.e., secondary metabolites, have naturally been a valuable source of various pharmacologically active compounds. The trivial functional annotations associated with plant extracts include antioxidant [1], anti-aging [2], anti-metastatic [3], anti-obesity [4], and antiparasitic [5] activities, finding applications in cosmetic and healthcare sectors. Notwithstanding the chemosystematics, the metabolomic state of a plant undergoes variations according to the transcriptome, proteome, and prevalent environmental conditions [6,7]. Additionally, plant chemodiversity is further enriched through the intricate biotransformation of metabolites into closely related derivatives, including methylated, acetylated, and/or glycosylated, generating a gamut of metabolic sub-classes [8]. Owing to the diverse metabolomes, the biochemical characterization of secondary metabolites in plants has been considered an arduous task. In the past, various extraction protocols, chromatographic systems, and spectrometric methods have provided an impetus to phytochemical studies [9,10].

Ushering in the metabolomics era, mass spectrometry (MS)-based metabolite characterization has evolved as an adept platform to discern chemotaxonomy [11], metabolic pathways [12], and phytochemical characterization [13], altogether complementing the -omics cascade of genomics and proteomics. Metabolomics enables an unbiased high-throughput screening and characterization of the overall gamut of metabolic entities through chromatographic separation, high-resolution MS, and enhanced detection sensitivity [14]. However, the subsequent functional annotation of the identified metabolites often seems difficult owing to the different titers of metabolic repertoire influencing the biological phenotypes [15]. Hence, the neoteric multi-parallel approaches need to be explored toward the expeditious screening and characterization of functional metabolites in diverse plant samples.

Historically, indigenous plants have been harnessed as food supplements, medicines, and cosmetics owing to their time-tested efficacies and traditional applications [16,17]. The family Betulaceae is a typical angiosperm distributed in the Northern hemisphere, with 6 genera and 170 species, mostly including trees or shrubs [18], and has customarily been used as plant ornaments, timbers, nut crops, and medicines [19]. Phytochemical analyses of Betulaceae plant extracts have revealed a rich composition of secondary metabolites, including terpenoids, phenolics, and flavonoids, rendering them functional properties, including anticancer [20], antioxidant [21], and antiviral [22]. Especially, the plants’ antioxidants, like vitamin C, flavonoids, and phenolic compounds, offer metabolic hotspots in ethnopharmacological studies.

Herein, we propose a metabolomic-cum-bioassay-guided approach aimed toward the systematic characterization of antioxidant metabolites from different biosystematic groups in the Betulaceae family. Further, the study aims to examine the effects of chemotaxonomic relatedness on the bioactivity phenotypes for the plant samples used in the study. We comprehensively employed MS-based metabolite profiling, solvent extraction, and preparatory-HPLC methods coupled with parallel bioassays to determine the specific metabolites conferring the desired antioxidant phenotypes.

## 2. Results

### 2.1. Metabolite Profiling and Antioxidant Activity Assay-Guided Screening of Plant Extracts

**Figure 1:**
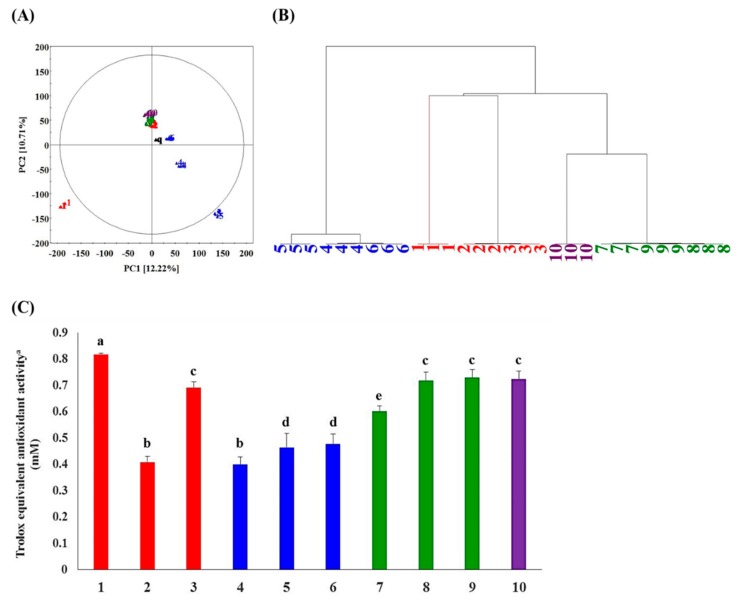
(**A**) Principal component analysis (PCA) score plot, (**B**) Hierarchical cluster analysis (HCA) dendrogram derived from the partial least squares discriminant analysis (PLS-DA) result, derived from positive mode data set of ultrahigh-performance liquid chromatography linear trap quadrupole-ion trap- mass spectrometry/mass spectrometry (UHPLC-LTQ-IT-MS/MS) of Betulaceae family plant extracts. (**C**) Antioxidant activities of 10 Betulaceae family plant extracts. Red color represents species belonging to the genus *Alnus* (1: *Alnus firma*, 2: *Alnus hirsuta*, and 3: *Alnus japonica*). Blue color represents species belonging to the genus *Betula* (4: *Betula schmidtii*, 5: *Betula dahurica*, and 6: *Betula pendula*). Green color represents species belonging to the genus *Carpinus* (7: *Carpinus cordata*, 8: *Carpinus turczaninowii*, and 9: *Carpinus laxiflora*). Purple color represents species belonging to the genus *Corylus* (10: *Corylus heterophylla*). The small letter on the bar graphs (a, b, c, d, and e) indicates significant differences from other values.

Metabolite profiling was performed to distinguish plants having antioxidant compounds among the 10 species of Betulaceae. Based on the principal component analysis (PCA) (Figure 1A) derived from the positive mode ultrahigh-performance liquid chromatography linear trap quadrupole-ion trap- mass spectrometry/mass spectrometry (UHPLC-LTQ-IT-MS/MS) datasets of 10 plant species, a clustered pattern depending on the plant genus was mostly observed, irrespective of their different times and areas of harvest. The analytical replicates of the spiked quality control (QC) samples were clustered at the center of the PCA score plot (Figure 1A), ensuring the normal performance of the instrument. We observed the clusters of metabolomic datasets for the genera *Alnus* (red color), *Carpinus* (green color), and *Corylus* (purple color) separated distinctly from the genus *Betula* (blue color). Notably*, Alnus firma* (*A. firma*) was separated from the cluster of the genus *Alnus* along PC1 (12.22%) and PC2 (10.71%). The hierarchical cluster analysis (HCA) dendrogram (Figure 1B) showed that the genus *Betula* and a group of other genera, *Alnus*, *Corylus*, and *Carpinus*, had two major branches. As the feature values used for the PCA score plot were based on the metabolomics data, the result indicated that phylogenetic factors influenced the chemical composition of samples rather than environmental factors.

The analyses of antioxidant activity (Figure 1C) for the Betulaceae family extracts show that five members, including *Alnus firma* (1), *A. japonica* (3), *Carpinus turczaninwii* (8), *Carpinus laxiflora* (9), and *Corylus heterophylla* (10), had significantly higher antioxidant activities compared to the rest of the members. Among them, *A. firma* (1) displayed the highest antioxidant activity. Considering the metabolite profiling data as well as the bioactivities, *A. firma* (1) extracts were screened for a further course of studies.

### 2.2. Effect of the Alnus firma Ethanol Extracts (AFEE) on H_2_O_2_-Induced HDF Cell Damage

**Figure 2:**
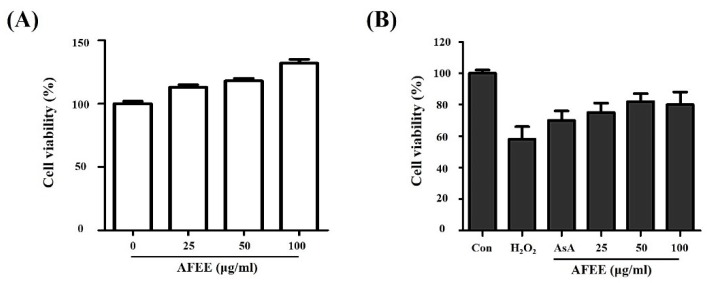
Assessment of protective ability against H_2_O_2_-induced Human Dermal Fibroblasts (HDF) cell damage by *A. firma* ethanol extract (AFEE). (**A**) Effect of AFEE on the viability of HDF cells. Cells incubated with different concentrations of AFEE for 24 h. Cell viability was measured by XTT assay. (**B**) Effect of AFEE on hydrogen peroxide-induced death of HDF cells. Con, negative control; AsA, ascorbic acid as positive control.

Assessment of protective ability against H_2_O_2_-induced HDF cell damage is one of the effective methods for evaluating the in vitro antioxidant activity of plant extracts. We determined the maximum non-toxic concentration for *A. firma* ethanol extracts (AFEE) on HDF cells, i.e., 100 μg/mL (Figure 2A), and performed experiments using doses below this critical concentration. Treatment of 1 mM hydrogen peroxide decreased the viability of HDF cells by 60% compared to the viability in the control. Pre-treatment with 50 μM ascorbic acid, used as the positive control, restored the viability of HDF cells compared to the hydrogen peroxide group. Ascorbic acid prevents oxidative damages in HDF cells. In line with this, pretreatment with AFEE (25, 50, and 100 μg/mL) resulted in the prevention of cell death induced by hydrogen peroxide (Figure 2B).

### 2.3. Bioactivity Assay for A. Firma SPE Fraction Eluates

**Figure 3:**
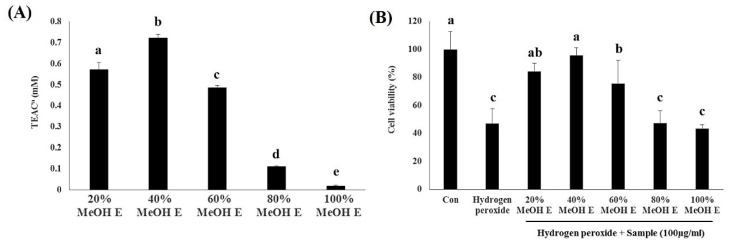
Bioassays for SPE MeOH eluates. (**A**) The ABTS radical scavenging activities. (**B**) Protective effects against H_2_O_2_-induced damage in HDF cells. The observed values were statistically tested using one-way analysis of variance (ANOVA). ^a^ Trolox equivalent antioxidant activity. The same letters (a, b, c, d, e) indicate values that are not significantly different by Duncan’s test.

SPE of *A. firma* with different proportions of methanol/water solvent (20%, 40%, 60%, 80%, and 100%) was performed to separate the metabolites based on their polarity. The sample dry yields for methanol SPE extracts were observed in the order of 20% > 40% > 60% > 100% > 80% (Table S1). Substances, such as carbohydrates, amino acids, and organic acids, are polar and have high molecular weights and were thought to be included in the most polar solvent (20% methanol), leading to the highest yield with 20% methanol SPE eluate (20% MeOH E). In contrast, as the solvents became non-polar, it appears that the yields were reduced because of dissolution of the non-polar and low molecular weight substances. We observed that the 40% methanol SPE eluate (40% MeOH E) exhibited the highest ABTS radical scavenging activities followed by 20% > 60% > 80% > 100% (Figure 3A). The cell viability for untreated control cells was reduced to 42% following the treatment. Conversely, pretreatment with SPE eluates, including 20%, 40%, and 60% MeOH E, significantly reversed the H_2_O_2_-induced damages to HDF cell viability. The different levels of biological activities imply that five different SPE eluates of *A. firma* MeOH E might contain dissimilar proportions or some components of bioactive compounds. In particular, the 40% MeOH E treatment had the highest protective effect against oxidative stress-induced cell damage, with an over 92% survival rate (Figure 3B). We presume that 40% MeOH E was abundant in antioxidant compounds, and hence reduced the H_2_O_2_-induced oxidative damage in HDF cells.

### 2.4. Metabolite Profiling of SPE MeOH Eluates of A. firma Extracts

**Figure 4:**
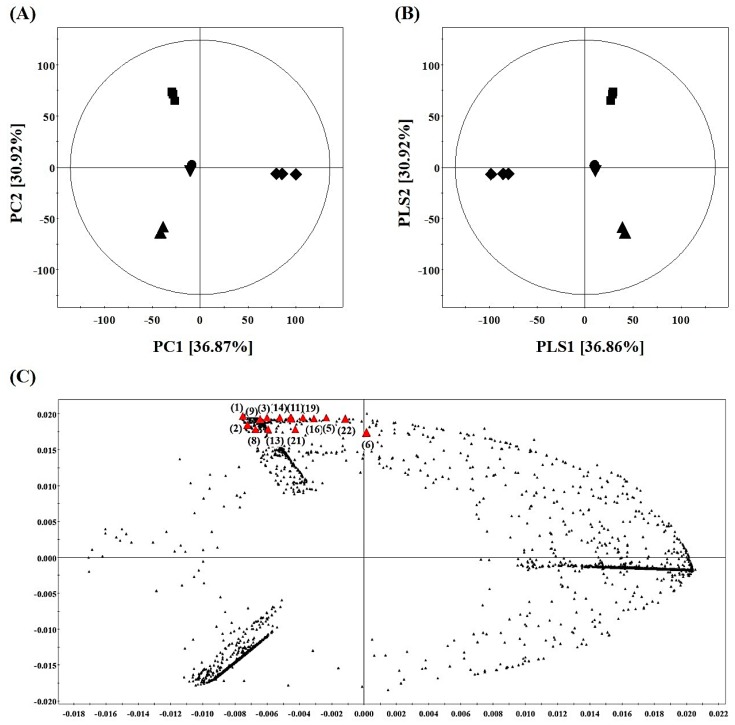
Multivariate analyses of the five SPE MeOH extracts of AFEE. (**A**) PCA score plot (R^2^X (0.812) and Q^2^ (0.698)) derived from the negative mode dataset of UHPLC-LTQ-IT-MS/MS. (**B**) PLS-DA score plot (R^2^X (0.825), R^2^Y (0.995), and Q^2^ (0.981)) derived from the negative mode dataset of UHPLC-LTQ-IT-MS/MS. Extracted by ● 20% MeOH, ■ 40% MeOH, ♦ 60% MeOH, ▲ 80% MeOH, and ▼ 100% MeOH. (**C**) The discriminant metabolites (red triangle) for 40% MeOH E are indicated in the loading plot, which were derived from the PLS-DA results. The numbers shown in the loading plot are as per Table 1.

Metabolite profiling using UHPLC-LTQ-IT-MS/MS combined with multivariate statistical analysis was conducted to analyze the discriminant metabolites among the five SPE MeOH eluates, including 20%, 40%, 60%, 80%, and 100%. In the PCA score plot derived from the negative mode data set (Figure 4A), the five SPE extracts were clustered separately from each other. Notably, the 40% MeOH E (■) was separated clearly from other extracts along PC2 (30.92%). The results showed that the chemical composition of each eluate was different, meaning that the complex substances in AFEE were separated according to the polarity in the SPE. Through the PLS-DA model (Figure 4B), overall, 48 variables were selected as significantly discriminant metabolites for five SPE MeOH eluates based on the variable importance in the projection (VIP) >0.7 and *p*-value < 0.05. Among them, 32 metabolites (19 flavonoids, 2 phenolic compounds, 8 diarylheptanoids, 2 stilbenoids, and 1 iridoid) were tentatively identified through a comparison of their mass fragmentation spectra and retention time with standard compounds, reference data, in-house library, and MassBank database (Table S2). With the PLS-DA model (Figure 4B), 14 metabolites discriminating the 40% MeOH E were determined and indicated on the loading plot derived from the PLS-DA results (Figure 4C). Among them, nine metabolites were tentatively identified and listed as follows (Table 1): (1) Ethyl gallate, (2) myricetin-3-O-galactoside, (5) myricetin-3-O-pentoside, (6) quercetin-3-O-glucoside, (9) quercetin-3-O-glucuronide, (11) hirsutoside, (14) luteolin-7-O-glucuronide, (16) pinosylvin diglucoside, and (22) platyphyllonol. Among them, (2) myricetin-3-O-galactoside has been previously reported in *A. firma*, while the other seven compounds have not been reported in *A. firma* yet, although they have been studied in other *Alnus* species [23]. The identified nine metabolites were classified into four categories including: (A) Flavonoids: (2) Myricetin-3-O-galactoside, (5) myricetin-3-O-pentoside, (6) quercetin-3-O-glucoside, (9) quercetin-3-O-glucuronide, and (14) luteolin-7-O-glucuronide; (B) phenolic acids: (1) Ethyl gallate; (C) stilbenoid: (16) Pinosylvin diglucoside; and (D) diarylheptanoids: (11) Hirsutoside and (22) platyphyllonol. Further, the identified flavonoids were categorized into flavonol and flavone glycosides.

### 2.5. Preparative HPLC Sub-Fractionation (15 and 31 Min) of 40% MeOH SPE Eluates and Antioxidant Assays

Preparative high-performance liquid chromatography (prep-HPLC) coupled with bioactivity assays was been a standard approach for target compounds analysis in plants. The 40% MeOH E was chosen for the analysis based on its highest antioxidant activity among the five SPE extracts obtained. Prep-HPLC for the 40% MeOH E in combination with antioxidant assays was performed to analyze the antioxidant potential of the prep-HPLC sub-fraction (Figure S1). During the prep-HPLC analysis, a total of 72 fractions were collected (1 min^−1^) and their antioxidant activities were assayed for the odd-numbered fractions. The highest antioxidant activities were recorded for the 15-min and 31-min prep-HPLC fractions, and hence, they were further subjected to UHPLC-LTQ-IT-MS/MS and UHPLC-Q-Orbitrap-MS analyses. A total of 14 metabolites were detected in the two fractions, of which, seven metabolites were tentatively identified (Table 2) as ethyl gallate (1), myricetin-3-O-galactoside (2), and quercetin-3-O-glucuronide (9) from the 15-min fraction; while, luteolin-7-O-glucuronide (14), pinosylvin diglucoside (16), platyphyllonol (22), and myricetin (51) were identified from the 31-min fraction.

**Figure 5:**
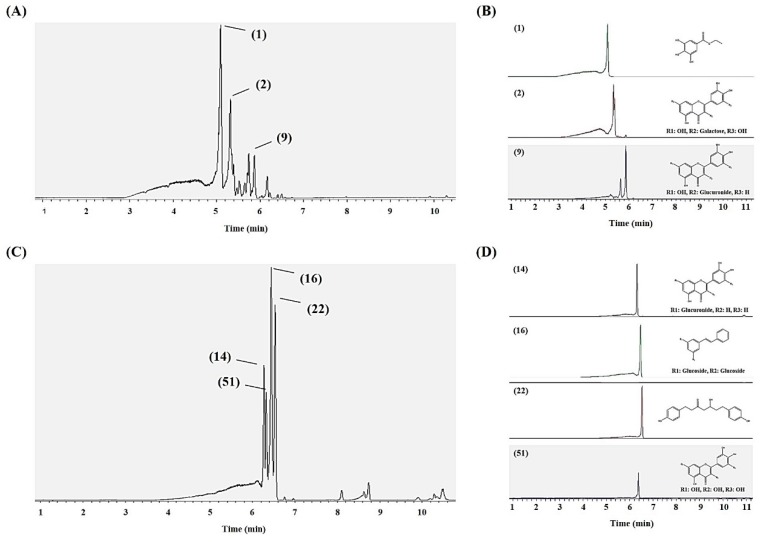
UHPLC-Q-Orbitrap-MS chromatograms. (**A**) 15-min and (**C**) 31-min, fractions. The ion-extracted chromatograms of identified metabolites of: (**B**) 15-min and (**D**) 31-min fractions, with structures of the putatively identified compounds.

Notably, six compounds were detected in total from both 40% SPE MeOH E, as well as its prep-HPLC sub-fractions (15 and 31 min), while myricetin (51) was detected only in the 31-min prep-HPLC sub fraction. The UHPLC-Q-Orbitrap-MS chromatogram, ion extracted chromatogram, and the structure of the identified metabolites of the 15- and 31-min fractions are shown in Figure 5.

### 2.6. Bioactivity Validation of Proposed Metabolites

**Figure 6:**
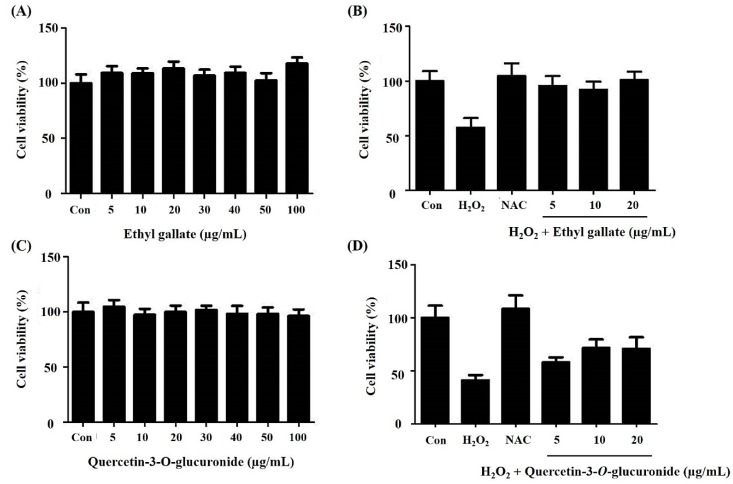
Effect of (**A**) ethyl gallate and (**C**) quercetin-3-*O*-glucuronide on HDF cell viability. Cells were incubated with different concentrations of ethyl gallate or quercetin-3-*O*-glucuronide for 24 h. Cell viability was measured using the XTT assay. Effect of (**B**) ethyl gallate and (**D**) quercetin-3-*O*-glucuronide on hydrogen peroxide-induced cell death in HDF cells. Con, negative control; NAC, n-acetylamine was used as the positive control.

Overall, 19 metabolites, including 9 overlapping compounds, were detected and proposed as antioxidant-related compounds based on the results of metabolite profiling and prep-HPLC analyses. The results suggested that several analytical methods can be applied to natural product research to detect a plethora of compounds. Of these, 10 compounds were tentatively identified. To validate whether the proposed metabolites had antioxidant properties, six commercial standard compounds were purchased, and confirmation experiments were performed. In the case of the ABTS radical scavenging activity assay, the EC50 values of the six commercial standard compounds were determined (Table 3). The observed EC50 values in order from the lowest to highest were: (1) Ethyl gallate, (9) quercetin-3-O-glucuronide, (2) myricetin-3-O-galactoside, (6) quercetin-3-O-glucoside, (14) luteolin-7-O-glucuronide, and (51) myricetin. In particular, ethyl gallate and quercetin-3-O-glucuronide displayed relatively high antioxidant activities, with no cytotoxicity even at the 100 μg/mL concentration as compared with other compounds (Figure 6A,C). Ethyl gallate and quercetin-3-glucuronide had cytoprotective effects against hydrogen peroxide-mediated cytotoxicity (Figure 6B,D). Notably, although the two compounds had similar levels of antioxidant activity in the ABTS assay, ethyl gallate effectively mitigated the oxidative damage within the non-cytotoxic concentration range compared to quercetin-3-O-glucuronide.

## 3. Discussion

We applied integrated metabolomics-cum-bio-assay-guided methodology to screen valuable plant resources, i.e., antioxidant secondary metabolites, from 10 species of the Betulaceae family. In general, the chemical composition of plants is affected by multiple factors, including their phylogeny, environment, climate, and metabolic processes. By applying these experimental protocols, we were able to understand what factors influence plant chemical composition more. However, since the plant samples analyzed in this research were the plant extracts mixed with leaves and stems, further study is needed to know the difference in chemical composition according to tissues. The compositions of plant secondary metabolites were dependent more on phylogeny rather than environmental factors [24]. However, some reports indicate that the data had little similarity with taxonomic patterns based on other traits, and the chemical profiles of some plant species may differ despite belonging to the same genus [25,26].

We evaluated the untargeted metabolite profiles and antioxidant activities from 10 species of the Betulaceae family and investigated whether the tendency of clustering formation acquired from chemotaxonomy was related their metabolite compositions. In general, we observed similar antioxidant activity levels for the ethanol extracts from the plants belonging to same genus groups; however, the *Alnus* genera extracts displayed significantly higher antioxidant levels compared to others. The results suggested that the variations in metabolite profiles for the species belonging to the same genus could manifest their chemotaxonomy and associated bioactivity. 

The *A. firma* (1) ethanol extract displayed the highest antioxidant activity coupled with a distant chemotaxonomic profile compared to the remaining nine species from the Betulaceae family. The antioxidant compounds in the *A. firma* extracts were able to mitigate the oxidative stress in the HDF cells, and thus enhance cell viability. According to the ‘free-radical theory’ [27], the cells are damaged by reactive oxygen stress (ROS), including superoxide anions, hydroxyl radicals, and hydrogen peroxide. The ROS produced via endogenous and exogenous sources damage DNA, fatty acids in lipids, and amino acids in proteins, leading to impaired physiological function and fast aging in cells. Consequently, the antioxidative defense mechanisms in live cells are the counteractions of the oxidative stress. The experimental results reveal the presence of strong antioxidants in the AFEE, which might have mitigated the oxidative stress in HDF cells, resulting in higher cell viability. As seen from the results, the tendency to scavenge free radicals and confer cyto-protection is related, suggesting that the antioxidants in the extracts were directly responsible for the reduced oxidative stress of HDF cells, as also proposed in the free-radical theory. 

Although *A. firma* was screened as a valuable plant with remarkable antioxidant activity, various separation and analytical methods were required to characterize the antioxidant compounds owing to its complex chemical composition. Separation of active compounds based on affinity is valuable, as the solid phase extraction (SPE) method offers rapid extraction with minimal labor, and yet, has high yield concentrations, and is therefore widely used as a pretreatment process for plant chemical analysis [28].

The metabolite profiling of SPE eluates of *A. firma* extracts indicated flavonoids, phenolic acids, stilbenoids, and diarylheptanoids as the main metabolite classes in the 40% MeOH E. Considering the glycosidic moieties, the glycoside forms of flavonoids are more highly polar than the corresponding aglycone forms, which complicates their extraction in highly polar solvents, such as 40% methanol [29]. Flavonoids are representative compounds of plant origin and are known to possess antioxidant activities through electron transfer to free radicals [30]. Ethyl gallate is a natural phytochemical produced by plants and possesses antioxidant properties [31]. Pinosylvin, a stilbenoid derivative, acts as an antioxidant by donating and accepting electrons [32]. Diarylheptanoids are compounds found mainly in plants of the Betulaceae family and have associated antioxidant properties [33]. Hirsutoside and platyphyllonol are linear diarylheptanoids with antioxidant activities, but they have been reported from other *Alnus* species [23] and not in *A. firma*. Hence, we infer that 40% MeOH SPE eluates were primarily composed of compounds with high antioxidant activities, affecting both the ABTS radical scavenging, as well as mitigating oxidative stress in HDF cells. Metabolite profiling is a method of analyzing significantly different metabolites using statistics, based on which it was possible to list 40% MeOH E-specific metabolites in this study. However, information was insufficient to clarify which metabolites are truly involved in the antioxidant effects. Therefore, we performed a preparative high-performance liquid chromatography (prep-HPLC) for further analysis. Prep-HPLC coupled with bioactivity assays are standard methods for analyzing target compounds in plants. These methods can be useful tools in the rapid search for functional bioactives in plants.

Ethyl gallate and quercetin-3-O-glucuronide were detected using both MS-based metabolite profiling and preparatory-HPLC methods coupled with parallel antioxidant assays. This highlights a strong linkage to the antioxidant activities of compounds from *A. firma*. Gallic acid has strong antioxidant activity due to its hydroxyl and carboxyl groups in the aromatic ring [34]. Although one hydroxyl group is ethylated, the structural skeleton may be consistent with the antioxidant activity of ethyl gallate (1). The antioxidant activity of quercetin-3-O-glucuronide (9) may be attributed to the quercetin aglycone itself, as well as the free carboxyl group in its glucuronide moiety with high polarity [35]. Considering the ‘free radical theory’, the two strongest ABTS radical scavengers were tested for their ability to confer protection against oxidative damage in HDF cells. Few studies have investigated this in ethyl gallate and quercetin-3-O-glucuronide. Kalaivani revealed that ethyl gallate, a nonflavonoid phenolic compound, is a potent antioxidant and functions as a radical scavenger, metal ion chelator, and hydrogen donor [36]. The glycosidic moiety of flavonoids affects the bioactivities by altering the molecular size, charge, and polarity. Generally, when flavonoids are ingested and enter the circulatory system, they are presented in the glucuronide form through enterohepatic circulation [37]. Our results support the possibility that ethyl gallate and quercetin-3-O-glucuronide in *A. firma* may contribute to antioxidant activity observed in the in vitro systems. This research provides a platform for elucidating the useful metabolites of plants. 

## 4. Materials and Methods 

### 4.1. Chemicals and Reagents

Ethanol, methanol, acetonitrile, and water were purchased from Fisher Scientific (Pittsburgh, PA, USA). Potassium persulfate, 2,2″-azinobis (3-ethylbenzothiazoline-6-sulfonic acid) diammonium salt (ABTS), 6-hydroxy-2,5,7,8-tetramethylchroman-2-carboxylic acid (Trolox), hydrogen peroxide, ascorbic acid, N-acetyl-L-cysteine (NAC), 2,3-Bis-(2-Methoxy-4-Nitro-5-Sulfophenyl)-2H-Tetrazolium-5-Carboxanilide (XTT), and standard compounds were purchased from Sigma-Aldrich (St. Louis, MO, USA). Dulbecco’s modified Eagle’s medium (DMEM), fetal bovine serum (FBS), penicillin-streptomycin, phosphate-buffered saline (PBS), and trypsin-Ethylenediaminetetraacetic acid (EDTA) were purchased from Gibco (Grand Island, NY, USA).

### 4.2. Sample Information and Preparation

Ten plant species belonging to the Betulaceae family were used in this study (Table 4). Plant samples were harvested between July and August 2014 from six provinces, one metropolitan city, and one special self-governing province of Korea. All voucher specimens were kept in the herbarium of the National Institute of Biological Resources (NIBR, Incheon, Korea). The above-ground plant samples were dried under shade, and each sample (100 g) was extracted with 70% ethanol (1000 mL). Further, each sample was concentrated using a rotary vacuum evaporator (Eyela, Tokyo, Japan) followed by filtration using Millex^®^ GP 0.22-μm filters (Merck Millipore, Billerica, MA, USA). The concentrated solutions were freeze-dried and stored in deep freezer (−70 °C) until analyses.

### 4.3. Metabolite Profiling of Plants from Betulaceae

The dried solvent extracts from 10 plant species belonging to the Betulaceae family were dissolved in 70% ethanol (20 mg/mL) and filtered through a 0.2-μm PTFE filter. The UHPLC-LTQ-IT-MS/MS analysis was performed using a Thermo Fisher Scientific LTQ XL linear ion trap mass spectrometer consisting of an electrospray interface (Thermo Fisher Scientific, San José, CA), coupled with a DIONEX UltiMate 3000 RS pump, RS autosampler, RS column compartment, and RS diode array detector (Dionex Corporation, Sunnyvale, USA). The sample analytes were separated on a Thermo Scientific Syncronis C18 UHPLC column (100 mm length × 2.1 mm i.d., 1.7-μm particle size). The mobile phase consisted of A (0.1% [v/v] formic acid in water) and B (0.1% [v/v] formic acid in acetonitrile). The solvent gradient flow was programmed as follows: 10% B for 0–1 min, 10–100% B for 1–15 min, 100% B for 15–18 min, 100–10% B for 18–19 min, and again 10% B for 19–22 min. The solvent flow rate was set at 0.3 mL/min. The sample injection volume was 10 μL. The temperature of the column was maintained at 35 °C. The detection wavelength for the photodiode array detector ranged from 200 to 600 nm. Ion trap was performed in the positive and negative full-scan ion modes within a range of 150 to 1000 m/z. The operating parameters were tuned as follows: Source voltage ± 5 kV; capillary voltage 39 V; capillary temperature 275 °C. Tandem MS analysis was performed using scan-type turbo data-dependent scanning (DDS) using the same conditions as used for MS scanning. Three spectrometric repetitions were applied to each sample. The UHPLC-LTQ-IT-MS/MS data were acquired with Xcalibur software version 2.00 (Thermo Fisher Scientific, San José, CA), and the raw data files were converted to NetCDF (*.cdf) format using Xcalibur software. After conversion, the NetCDF files were subjected to preprocessing, correction of retention time and baseline, and peak extraction using the MetAlign software package (http://www.metalign.nl). The resulting data were exported to a Microsoft Excel file (Microsoft, Redmond, WA, USA). The multivariate statistical analysis was carried out using SIMCA-P+ 12.0 software (Umetrics, Umea, Sweden). The PCA, PLS-DA, and HCA were performed to analyze the metabolomic profiles and chemotaxonomy of the plants.

### 4.4. Solid-Phase Extraction (SPE) for A. Firma Extracts

The freeze-dried *A. firma* extract (1 g) was dissolved in 70% ethanol (700 μL). The C18 Sep-Pak cartridge (Waters, Milford, MA, USA) was pre-washed with 50 mL of 20% methanol. The sample analyte was loaded on to the cartridge and extracted using 50 mL of each solvent in the following order: 20%, 40%, 60%, 80%, and 100% methanol. Each eluate from the fractionation was dried using a speed vacuum concentrator and stored in a −70 °C freezer until analyses. The dried yields of five extracts from *A. firma* are shown in the Table S1.

### 4.5. Metabolite Profiling and Multivariate Analysis of A. Firma SPE Samples

The dried SPE samples from *A. firma* were dissolved in 70% ethanol (5 mg/mL) and filtered using a 0.2-μm PTFE filter prior to UHPLC-LTQ-IT-MS/MS analysis. The instrumental condition and data processing methods were similar to those described in the previous section. The significantly different compounds in the SPE samples were selected based on the variable importance in the projection (VIP) > 0.7, and the analysis of variance (ANOVA), tested at *p*-value < 0.05. The putatively identified compounds were confirmed using authentic standard compounds through a comparison of both the mass spectra (MS) and retention time (RT) data. In cases where standard compounds were not available, tentative identification was performed based on the MS data from the National Institute of Standards and Technology (NIST, Gaithersburg, MD, USA, 2005), combined chemical dictionary (CCD) version 7.2 (Chapman & Hall/CRC), and published literature [23].

### 4.6. Bioactivity Assays

#### 4.6.1. ABTS Radical Scavenging Assay of Plant Extracts Obtained from the Betulaceae Family

The antioxidant levels of plant extracts from the Betulaceae family were analyzed using a modified ABTS radical scavenging activity assay [38]. The dried extracts from each sample were dissolved in 70% ethanol (1 mg/mL). ABTS ammonium was dissolved in 2.45 mM potassium persulfate solution (7 mM). The ABTS stock solution was maintained at room temperature for one day in order to allow it to turn into a dark blue solution. Before assaying, the ABTS stock solution was diluted with distilled water until the OD 734 nm value reached 0.7. The reaction mixtures containing 10 μL of the dissolved sample and 190 μL of ABTS solution were incubated at room temperature in a 96-well microtiter plate. After 6 min, the OD 734 nm values were recorded using a microplate reader (BioTek ELx808, Winooski, VT, USA), with 6-hydroxy-2,5,7,8-tetramethylchroman-2-carboxylic acid (trolox) used as a standard compound. The concentration of trolox solution ranged from 0.0625 to 0.5 mM. The antioxidant activity of the sample extracts is represented as trolox equivalent antioxidant capacity (TEAC, mM). The significant differences were analyzed using analysis of variance (ANOVA). All experiments were carried out in triplicate.

#### 4.6.2. ABTS Radical Scavenging Assay for SPE Eluates of Extracts from *A. firma*

Each dried SPE eluate from *A. firma* extract was dissolved in 70% ethanol (0.1 mg/mL) and subjected to ABTS radical scavenging assay in triplicate using the method described above.

### 4.7. Cell Culture and Maintenance

HDF cell lines were procured from the American Type Culture Collection (Manassas, VA, USA). The HDF cells were cultured in DMEM medium supplemented with 10% heat inactivated FBS, 2 mM glutamate, 100 U/mL penicillin, and 100 μg/mL streptomycin. The cells were maintained at 37 °C in a 5% CO^2^ incubator. The cells were cultured to approximately 80% confluence, harvested with 0.25% trypsin-EDTA, and further sub-cultured for an additional 48 h in DMEM. 

#### XTT Cell Viability Assay using Extracts of *A. firma* Plants

The HDF cell proliferation rates were analyzed using 2,3-bis(2-methoxy-4-nitro-5-sulfophenyl)-2H-tetrazolium-5-carboxanilide inner salt (XTT) assay (WelGene, Seoul, Korea). The actively growing log phase HDF cells were seeded in 96-well plates (1 × 104 cells/well) for 24 h. The cells were divided as control and treatment groups subjected to the concentrations indicated. After 24 h, the number of viable cells were determined using XTT assay. The protective effects of *A. firma* ethanol extracts (AFEE) against hydrogen peroxide-induced cell death were evaluated using seeding HDF cells (5 × 10^4^ cells/well) cells in 12-well plates. After 48 h of pre-incubation, the cells were treated with various concentrations (25, 50, and 100 μg/mL) of AFEE, followed by addition of 1 mM hydrogen peroxide to each well. After 3 h of incubation of the treated groups, the % cell viability was determined using XTT assay. To determine the effects of SPE eluates on hydrogen peroxide-induced cell death, HDF cells were seeded in 12-well plates. After 48 h of incubation, the cells were treated with 100 μg/mL of each SPE eluate, followed by addition of 1 mM hydrogen peroxide to each well. After 3 h of incubation, the number of viable cells was determined using XTT assay. The appropriate negative (solvent) and positive (50 μM ascorbic acid) controls were maintained during each bioassay. Images of the cells were collected using a light microscopy set to 40-fold magnification. Absorbance (A) was determined with an enzyme calibrator at 450 nm. The % cell viability was determined using the following formula:

(1) Cell viabilityXTT =(A450 nm Treated groupsA450 nm Control groups)×100%

### 4.8. Combined Preparative HPLC Analysis and ABTS Assay of 40% SPE Eluates of A. Firma Extracts

The 40% methanol SPE eluates (40% MeOH E) were further subjected to preparative high-performance liquid chromatography (prep-HPLC). The prep-HPLC system was fitted with a diode array detector (PDA) L-2455, a binary pump L-2130 (Hitachi, Tokyo, Japan), and a YMC-Pack pro C18 column (250 mm length × 4.6 mm i.d., 5-μm particle size). The mobile phases consisted of A (5% acetonitrile in water) and B (100% acetonitrile). The solvent gradients were programmed as follows: 100% A for 0–2 min, 0–40% B for 2–62 min, 40–100% B for 62–67 min, and 0–100% A for 67–72 min. Before performing the prep-HPLC, 1 mg/mL of 40% MeOH E was injected for HPLC profiling. The flow rate was set at 1 mL/min and the PDA was tuned to range from 220 to 600 nm. Following HPLC profiling, 40 mg/mL of 40% MeOH E was injected for prep-HPLC with a flow rate of 1 mL/min and the absorption wavelength was set at 220 nm. Overall, 72 sub-fractions were collected at a rate of 1 fraction per minute. The prep-HPLC sub-fractions were dried using a speed vacuum concentrator and stored in a freezer (−70 °C) until further analyses. The dried fractions were dissolved in 70% ethanol (0.1 mg/mL) and ABTS radical scavenging activity assays were performed as described in the previous section.

### 4.9. Analysis of Bioactive Prep-HPLC Sub-Fractions Using UHPLC-LTQ-IT-MS/MS and UHPLC-Q-Orbitrap-MS

The selected bioactive sub-fractions from prep-HPLC were further subjected to UHPLC-LTQ-IT-MS/MS and UHPLC-quadrupole-orbitrap MS (Q-Orbitrap-MS) analyses. In the case of UHPLC-LTQ-IT-MS/MS, the instrument operating conditions were similar to those described in the previous section. In case of UHPLC-Q-Orbitrap-MS, a Dionex Ultimate 3000 UHPLC system (Thermo Fisher Scientific, Bremen, Germany) coupled to the Q-Exactive Orbitrap MS (Thermo Fisher Scientific, Bremen, Germany) was used. The UHPLC system was equipped with an Ultimate 3000 RS pump, Ultimate 3000 RS column compartment, and an Ultimate 3000 RS autosampler, all operated using Dionex Chromeleon software (Version 6.8, Dionex, Sunnyvale, CA, USA). The Hypersil Gold C18 selectivity LC column (50 mm length × 2.1 mm i.d., 1.9 μm particle size; Thermo Fisher scientific, Waltham, MA, USA) was used with acetonitrile (mobile phase A), water (mobile phase B) containing 0.1% formic acid, and at a flow rate of 0.3 mL/min. The gradient program was run as follows: 100% B for 20 min, then to 100% A in 0.5 min. The column was re-equilibrated at 100% B for 2.5 min prior to the next injection. The total run time for each injection was 23 min. The injection volume was 10 µL. The column oven temperature and autosampler temperature were set to 25 and 4 °C, respectively.

The Q-Exactive Orbitrap MS equipped with a heated electrospray ionization source (HESI) was operated in the negative ion mode. MS was operated in the full-scan mode. The spray voltage (negative mode), capillary temperature, probe heater temperature, and S-lens RF level were set to 3.3 kV, 320 °C, 300 °C, and 60, respectively. The sheath gas and auxiliary gas flow rates were set to 30 and 10 L/min, respectively. Nitrogen was used for spray stabilization, for collision-induced dissociation in HCD cells, and damping gas in the C-trap. The following parameters were used in full MS scan mode: Resolution 35,000 FWHM, scan range 100–1000 m/z, and the automatic gain control (AGC) target was set at 1e^6^ with a maximum injection time (IT) of 100 ms. The instrumental device was calibrated in the negative and positive mode once a week using the manufacturer’s calibration solutions.

## 5. Conclusions

In this study, we proposed a strategy for screening useful plant resources and identifying bioactive compounds based on metabolomics involving a combination of two analytical methods. Our results reveal that the two analytical approaches may cover a wide range of detected metabolites. Through this analysis, we were able to select useful plant species, track their bioactive compounds, and isolate ethyl gallate and quercetin-3-O-glucuronide as antioxidant compounds in *A. firma*. This research provides a metabolomic-cum-bioassay-guided methodology toward the comprehensive analyses and characterization of bioactive plant metabolites.

## Figures and Tables

**Table 1:** Tentatively identified metabolites in a 40% MeOH SPE eluate (40% MeOH E) based on metabolite profiling and multivariate statistical analysis.

No. ^a^	Putative Identification ^b^	UHPLC-LTQ-IT-MS/MS					
		RT ^c^ (min)	m/z Nega ^d^	m/z Posi ^e^	M.W. ^f^	MS^n^ Fragment Pattern	UV(nm)
1	Ethyl gallate	6.72	197	199	198	159 [posi] ^g^	217
2	Myricetin-3-*O*-galactoside	7.04	479	481	480	319 [posi]	215,357
3	N.I. 1	7.24	343	345	344	269	247
5	Myricetin-3-*O*-pentoside	7.44	449	451	450	319 [posi]	218,355
6	Quercetin-3-*O*-glucoside	7.57	463	465	464	301 > 179, 151	207,255
8	N.I. 2	7.72	413	415	414	311	-
9	Quercetin-3-*O*-glucuronide	7.73	477	479	478	301	-
11	Hirsutoside	8.02	491	493	492	311	-
13	N.I. 3	8.03	537	-	-	-	-
14	Luteolin-7-*O*-glucuronide	8.22	461	463	462	285, 267	-
16	Pinosylvin diglucoside	8.27	581[M+FA]^−^	537	536	375 > 213 [posi]	214,274
19	N.I. 6	8.47	325[M+FA]^−^	303[M + Na]^+^	280	113	-
21	N.I. 7	8.74	327	329	328	-	-
22	Platyphyllonol	8.79	313	315	314	297 [posi]	205,366

^a^ Number is based on the Table S2. ^b^ Putative metabolites based on VIP > 0.7 and *p*-value < 0.05. ^c^ Retention time. ^d^ Ion detected in negative mode. ^e^ Ion detected in positive mode. ^f^ Molecular weight. ^g^ MS^n^ fragment patterns detected in positive mode. N.I: Non-identified metabolite.

**Table 2:** Tentatively identified metabolites from 15-min and 31-min prep-HPLC sub-fractions of the 40% MeOH SPE eluate.

Fractions	No ^a^	Putative Identification	UHPLC-LTQ-IT-MS/MS						UHPLC-Q-Orbitrap-MS				ID ^h^
			RT ^b^ (min)	m/z Nega ^c^	m/z Posi ^d^	M.W. ^e^	MS^n^ Fragment Pattern	UV (nm)	RT (min)	m/z Nega	M.F.b ^g^	Δppm	
15 min	1	Ethyl gallate	6.59	197	199	198	159 [posi] ^f^	217	5.10	197.0449	C_9_H_10_O_5_	2.2	STD
	2	Myricetin-3-*O*-galactoside	6.95	479	481	480	319 [posi]	215,357	5.34	479.0837	C_21_H_20_O_13_	–0.3	STD
	3	N.I. 1	7.17	343	345	344	269	247	-	-	-	-	-
	8	N.I. 2	7.60	413	415	414	311	-	-	-	-	-	-
	9	Quercetin-3-*O*-glucuronide	7.60	477	479	478	301	-	5.87	477.0685	C_21_H_18_O_13_	–0.2	STD
31 min	14	Luteolin-7-*O*-glucuronide	8.08	461	463	462	285, 267	-	6.32	461.0734	C_21_H_18_O_12_	0.2	STD
	16	Pinosylvin diglucoside	8.13	581 [M+COOH]^−^	537	536	375 > 213 [posi]	214,274	6.44	581.1887	C_26_H_32_O_12_	–0.8	CCD
	21	N.I. 7	8.61	327	329	328	-	-	-	-	-	-	-
	22	Platyphyllonol	8.64	313	315	314	297 [posi]	205,366	6.53	313.1449	C_19_H_22_O_4_	0.5	CCD
	49	N.I. 17	8.36	447	449	448	317	-	-	-	-	-	-
	50	N.I. 18	8.53	405	407	406	303	295	-	-	-	-	-
	51	Myricetin	8.97	317	319	318	289	-	6.38	317.0669	C_15_H_10_O_8_	–0.2	STD
	52	N.I. 19	8.97	287	289	288	153 [posi]	-	-	-	-	-	-
	53	N.I. 20	7.94	542	-	-	466	215,247,362	-	-	-	-	-

^a^ Number is based on Table S2. Newly detected metabolites are numbered from 49 to 53. ^b^ Retention time. ^c^ Ion detected in negative mode. ^d^ Ion detected in positive mode. ^e^ Molecular weight. ^f^ MS^n^ fragment patterns detected in positive mode. ^g^ Molecular formula. ^h^ Identification.

**Table 3:** Assessment of antioxidant activity of the proposed metabolites.

Compounds	EC_50_ (µg/mL)	Regression Curve	*R^2^*
Ethyl gallate (1)	61.6	*y* = –0.0048*x* + 0.6745	0.9872
Quercetin-3-*O*-glucuronoide (9)	64.6	*y* = −0.0048*x* + 0.6745	0.9872
Myricein-3-*O*-galactoside (2)	144.9	*y* = −0.0024*x* + 0.7120	0.9933
Quercetin-3-*O*-glucoside (6)	176.1	*y* = −0.0019*x* + 0.6958	0.9953
Luteolin-7-*O*-glucuronide (14)	215.3	*y* = −0.0016*x* + 0.6913	0.9959
Myricetin (51)	221.3	*y* = −0.0016*x* + 0.7154	0.9997

**Table 4:** Sample information of plants from the Betulaceae family used in the study.

No.	Family	Genus	Species	Collection Area	Collection Date
1	Betulaceae	*Alnus*	*firma*	Sin-ri, Goryeong-eup, Goryeong-gun, Gyeongsangbuk-do	2014-07-23
2	Betulaceae	*Alnus*	*hirsuta*	Sangjung-ri, Geumgwang-myeon, Anseong-si, Gyeonggi-do	2014-07-25
3	Betulaceae	*Alnus*	*japonica*	Yonggi-ri, Gibuk-myeon, Buk-gu, Pohang-si, Gyeongsangbuk-do	2014-07-30
4	Betulaceae	*Betula*	*schmidtii*	Icheon-ri, Sangbuk-myeon, Ulju-gun, Ulsan	2014-08-01
5	Betulaceae	*Betula*	*dahurica*	Ungyo-ri, Bangnim-myeon, Pyeongchang-gun, Gangwon-do	2014-08-08
6	Betulaceae	*Betula*	*pendula*	Sogye-ri, Hwanggan-myeon, Yeongdong-gun, Chungcheongbuk-do	2014-08-14
7	Betulaceae	*Carpinus*	*cordata*	Apgok-ri, Bongsan-myeon, Hapcheon-gun, Gyeongsangnam-do	2014-07-24
8	Betulaceae	*Carpinus*	*turczaninowii*	Jiro-ri, Byeongyeong-myeon, Gangjin-gun, Jeollanam-do	2014-08-12
9	Betulaceae	*Carpinus*	*laxiflora*	Seonheul-ri, Jocheon-eup, Jeju-si, Jeju special self-governing province	2014-08-24
10	Betulaceae	*Corylus*	*heterophylla*	Apgok-ri, Bongsan-myeon, Hapcheon-gun, Gyeongsangnam-do	2014-07-24

## Supplementary Materials

The following are available online at https://www.mdpi.com/2218-1989/9/9/186/s1, Figure S1: (**A**) HPLC profiling chromatogram of 40% MeOH extract and (**B**) ABTS radical scavenging activities of odd-numbered preparative HPLC fractions. ^a^ Trolox equivalent antioxidant capacity. Table S1: The dried yields of five extracts from *A. firma,* Table S2: Tentatively identified metabolites in five SPE extracts based on metabolite profiling combined with multivariate statistical analysis.
