# Supplementary material for: Untargeted Metabolomics Toward Systematic Characterization of Antioxidant Compounds in Betulaceae Family Plant Extracts
Source: Metabolites. 2019 Sep 16;9(9):186. doi: 10.3390/metabo9090186 (PMC6780370; doi:10.3390/metabo9090186)
Supplement: Supplementary file 1 [file metabolites-09-00186-s001.zip › metabolites-594199-SI/metabolites-594199-supplementary.pptx]

## Slide 1
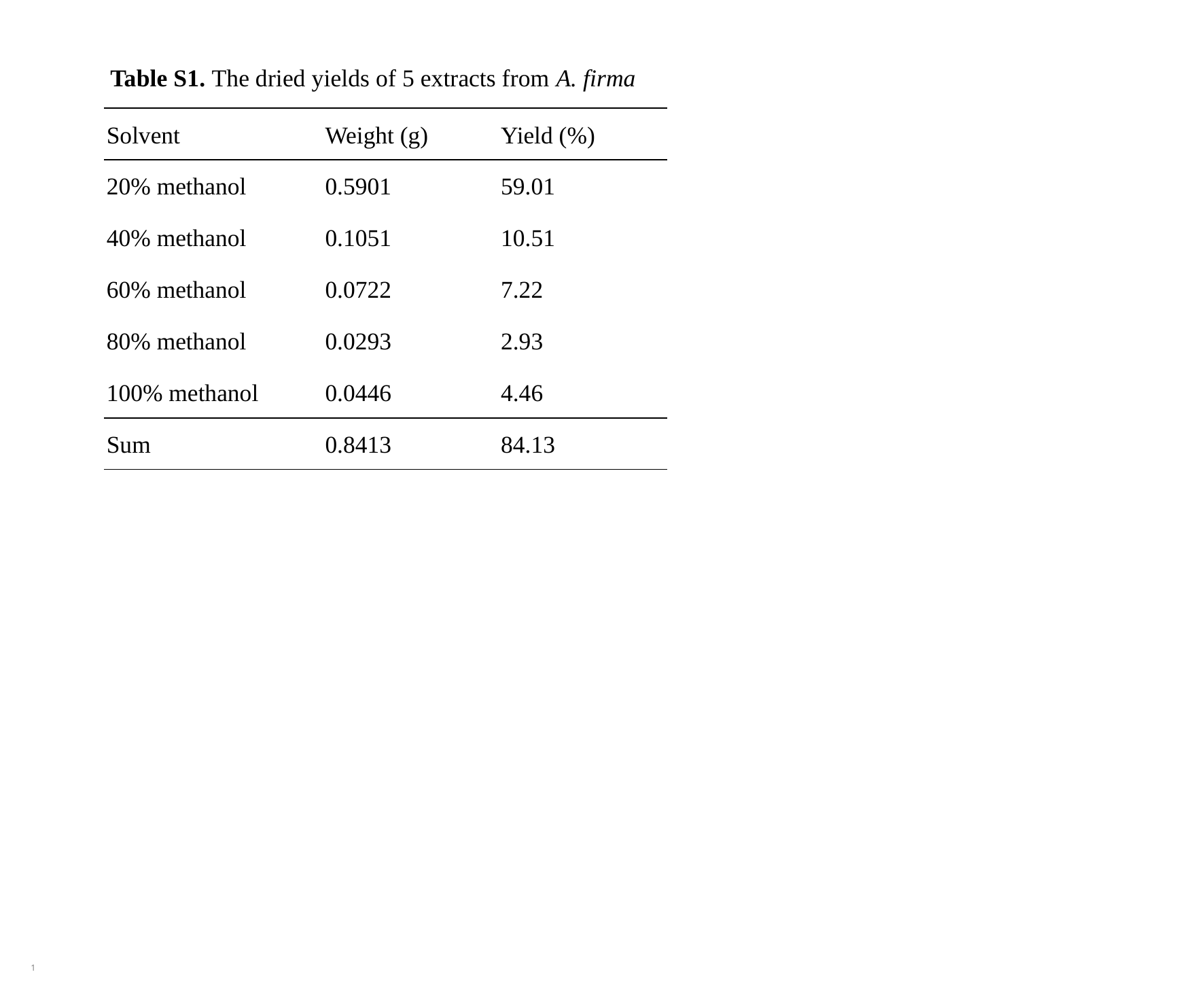

Table S1. The dried yields of 5 extracts from A. firma
| Solvent | Weight (g) | Yield (%) |
| --- | --- | --- |
| 20% methanol | 0.5901 | 59.01 |
| 40% methanol | 0.1051 | 10.51 |
| 60% methanol | 0.0722 | 7.22 |
| 80% methanol | 0.0293 | 2.93 |
| 100% methanol | 0.0446 | 4.46 |
| Sum | 0.8413 | 84.13 |

## Slide 2
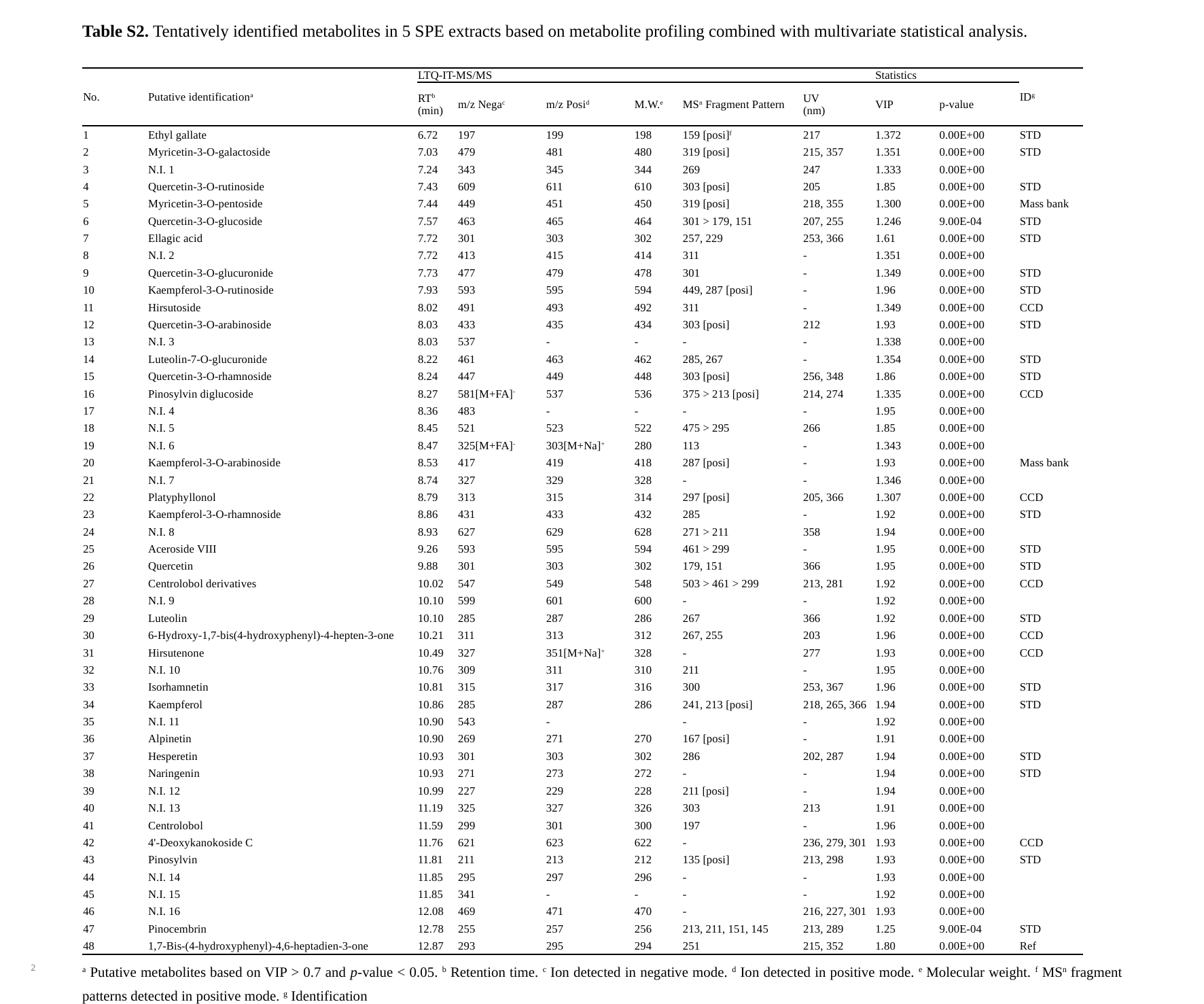

Table S2. Tentatively identified metabolites in 5 SPE extracts based on metabolite profiling combined with multivariate statistical analysis.
| No. | Putative identificationa | LTQ-IT-MS/MS | | | | | | Statistics | | IDg |
| --- | --- | --- | --- | --- | --- | --- | --- | --- | --- | --- |
| | | RTb (min) | m/z Negac | m/z Posid | M.W.e | MSn Fragment Pattern | UV (nm) | VIP | p-value | |
| 1 | Ethyl gallate | 6.72 | 197 | 199 | 198 | 159 [posi]f | 217 | 1.372 | 0.00E+00 | STD |
| 2 | Myricetin-3-O-galactoside | 7.03 | 479 | 481 | 480 | 319 [posi] | 215, 357 | 1.351 | 0.00E+00 | STD |
| 3 | N.I. 1 | 7.24 | 343 | 345 | 344 | 269 | 247 | 1.333 | 0.00E+00 | |
| 4 | Quercetin-3-O-rutinoside | 7.43 | 609 | 611 | 610 | 303 [posi] | 205 | 1.85 | 0.00E+00 | STD |
| 5 | Myricetin-3-O-pentoside | 7.44 | 449 | 451 | 450 | 319 [posi] | 218, 355 | 1.300 | 0.00E+00 | Mass bank |
| 6 | Quercetin-3-O-glucoside | 7.57 | 463 | 465 | 464 | 301 > 179, 151 | 207, 255 | 1.246 | 9.00E-04 | STD |
| 7 | Ellagic acid | 7.72 | 301 | 303 | 302 | 257, 229 | 253, 366 | 1.61 | 0.00E+00 | STD |
| 8 | N.I. 2 | 7.72 | 413 | 415 | 414 | 311 | - | 1.351 | 0.00E+00 | |
| 9 | Quercetin-3-O-glucuronide | 7.73 | 477 | 479 | 478 | 301 | - | 1.349 | 0.00E+00 | STD |
| 10 | Kaempferol-3-O-rutinoside | 7.93 | 593 | 595 | 594 | 449, 287 [posi] | - | 1.96 | 0.00E+00 | STD |
| 11 | Hirsutoside | 8.02 | 491 | 493 | 492 | 311 | - | 1.349 | 0.00E+00 | CCD |
| 12 | Quercetin-3-O-arabinoside | 8.03 | 433 | 435 | 434 | 303 [posi] | 212 | 1.93 | 0.00E+00 | STD |
| 13 | N.I. 3 | 8.03 | 537 | - | - | - | - | 1.338 | 0.00E+00 | |
| 14 | Luteolin-7-O-glucuronide | 8.22 | 461 | 463 | 462 | 285, 267 | - | 1.354 | 0.00E+00 | STD |
| 15 | Quercetin-3-O-rhamnoside | 8.24 | 447 | 449 | 448 | 303 [posi] | 256, 348 | 1.86 | 0.00E+00 | STD |
| 16 | Pinosylvin diglucoside | 8.27 | 581[M+FA]- | 537 | 536 | 375 > 213 [posi] | 214, 274 | 1.335 | 0.00E+00 | CCD |
| 17 | N.I. 4 | 8.36 | 483 | - | - | - | - | 1.95 | 0.00E+00 | |
| 18 | N.I. 5 | 8.45 | 521 | 523 | 522 | 475 > 295 | 266 | 1.85 | 0.00E+00 | |
| 19 | N.I. 6 | 8.47 | 325[M+FA]- | 303[M+Na]+ | 280 | 113 | - | 1.343 | 0.00E+00 | |
| 20 | Kaempferol-3-O-arabinoside | 8.53 | 417 | 419 | 418 | 287 [posi] | - | 1.93 | 0.00E+00 | Mass bank |
| 21 | N.I. 7 | 8.74 | 327 | 329 | 328 | - | - | 1.346 | 0.00E+00 | |
| 22 | Platyphyllonol | 8.79 | 313 | 315 | 314 | 297 [posi] | 205, 366 | 1.307 | 0.00E+00 | CCD |
| 23 | Kaempferol-3-O-rhamnoside | 8.86 | 431 | 433 | 432 | 285 | - | 1.92 | 0.00E+00 | STD |
| 24 | N.I. 8 | 8.93 | 627 | 629 | 628 | 271 > 211 | 358 | 1.94 | 0.00E+00 | |
| 25 | Aceroside VIII | 9.26 | 593 | 595 | 594 | 461 > 299 | - | 1.95 | 0.00E+00 | STD |
| 26 | Quercetin | 9.88 | 301 | 303 | 302 | 179, 151 | 366 | 1.95 | 0.00E+00 | STD |
| 27 | Centrolobol derivatives | 10.02 | 547 | 549 | 548 | 503 > 461 > 299 | 213, 281 | 1.92 | 0.00E+00 | CCD |
| 28 | N.I. 9 | 10.10 | 599 | 601 | 600 | - | - | 1.92 | 0.00E+00 | |
| 29 | Luteolin | 10.10 | 285 | 287 | 286 | 267 | 366 | 1.92 | 0.00E+00 | STD |
| 30 | 6-Hydroxy-1,7-bis(4-hydroxyphenyl)-4-hepten-3-one | 10.21 | 311 | 313 | 312 | 267, 255 | 203 | 1.96 | 0.00E+00 | CCD |
| 31 | Hirsutenone | 10.49 | 327 | 351[M+Na]+ | 328 | - | 277 | 1.93 | 0.00E+00 | CCD |
| 32 | N.I. 10 | 10.76 | 309 | 311 | 310 | 211 | - | 1.95 | 0.00E+00 | |
| 33 | Isorhamnetin | 10.81 | 315 | 317 | 316 | 300 | 253, 367 | 1.96 | 0.00E+00 | STD |
| 34 | Kaempferol | 10.86 | 285 | 287 | 286 | 241, 213 [posi] | 218, 265, 366 | 1.94 | 0.00E+00 | STD |
| 35 | N.I. 11 | 10.90 | 543 | - | | - | - | 1.92 | 0.00E+00 | |
| 36 | Alpinetin | 10.90 | 269 | 271 | 270 | 167 [posi] | - | 1.91 | 0.00E+00 | |
| 37 | Hesperetin | 10.93 | 301 | 303 | 302 | 286 | 202, 287 | 1.94 | 0.00E+00 | STD |
| 38 | Naringenin | 10.93 | 271 | 273 | 272 | - | - | 1.94 | 0.00E+00 | STD |
| 39 | N.I. 12 | 10.99 | 227 | 229 | 228 | 211 [posi] | - | 1.94 | 0.00E+00 | |
| 40 | N.I. 13 | 11.19 | 325 | 327 | 326 | 303 | 213 | 1.91 | 0.00E+00 | |
| 41 | Centrolobol | 11.59 | 299 | 301 | 300 | 197 | - | 1.96 | 0.00E+00 | |
| 42 | 4'-Deoxykanokoside C | 11.76 | 621 | 623 | 622 | - | 236, 279, 301 | 1.93 | 0.00E+00 | CCD |
| 43 | Pinosylvin | 11.81 | 211 | 213 | 212 | 135 [posi] | 213, 298 | 1.93 | 0.00E+00 | STD |
| 44 | N.I. 14 | 11.85 | 295 | 297 | 296 | - | - | 1.93 | 0.00E+00 | |
| 45 | N.I. 15 | 11.85 | 341 | - | - | - | - | 1.92 | 0.00E+00 | |
| 46 | N.I. 16 | 12.08 | 469 | 471 | 470 | - | 216, 227, 301 | 1.93 | 0.00E+00 | |
| 47 | Pinocembrin | 12.78 | 255 | 257 | 256 | 213, 211, 151, 145 | 213, 289 | 1.25 | 9.00E-04 | STD |
| 48 | 1,7-Bis-(4-hydroxyphenyl)-4,6-heptadien-3-one | 12.87 | 293 | 295 | 294 | 251 | 215, 352 | 1.80 | 0.00E+00 | Ref |
a Putative metabolites based on VIP > 0.7 and p-value < 0.05. b Retention time. c Ion detected in negative mode. d Ion detected in positive mode. e Molecular weight. f MSn fragment patterns detected in positive mode. g Identification

## Slide 3
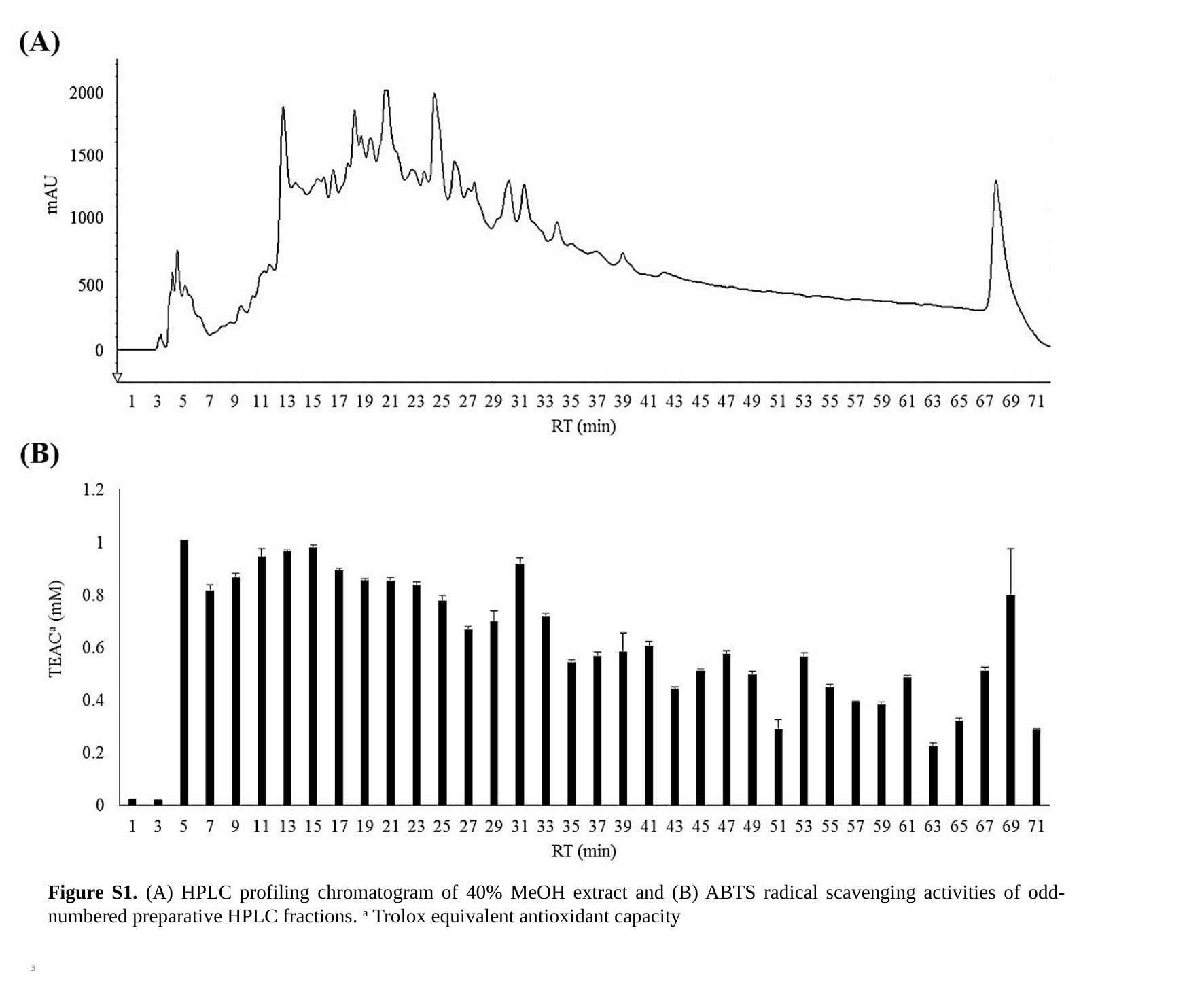

Figure S1. (A) HPLC profiling chromatogram of 40% MeOH extract and (B) ABTS radical scavenging activities of odd-numbered preparative HPLC fractions. a Trolox equivalent antioxidant capacity
